# Cannabis flower extracts as a multi-target agent in Alzheimer’s disease: Inhibition of AChE, BChE, and BACE1 with cell-protective effects in a PC12 cell model

**DOI:** 10.1186/s42238-026-00479-9

**Published:** 2026-07-31

**Authors:** Asif Shitrit, Ali Khattib, Soliman Khatib

**Affiliations:** 1 Natural Compounds and Analytical Chemistry Laboratory, MIGAL – Galilee Research Institute, P.O. Box 831, Kiryat Shmona, 11016 Israel; 2 Department of Biotechnology, Tel-Hai University of Kiryat Shmona in the Galilee, Tel-Hai, Kiryat Shmona, 1220800 Israel

**Keywords:** Alzheimer's disease, Cannabis (*Cannabis sativa L.*), Acetylcholinesterase, Butyrylcholinesterase, Beta-secretase, Hydrogen peroxide and Amyloid beta, Multi target therapy, Neuroprotection

## Abstract

Alzheimer’s disease (AD) is a neurodegenerative disorder marked by progressive cognitive decline. Despite extensive research, its multifactorial nature continues to challenge the development of effective therapies. Current treatments, including anticholinergic agents and amyloid β–targeted drugs, alleviate symptoms but fail to halt disease progression and often produce side effects. Therefore, novel multi-target strategies are urgently needed.

Cannabis (*Cannabis sativa L.*) has long been recognized for its medicinal properties, yet its potential in AD therapy has only recently gained attention. Previous studies have mainly focused on single cannabinoids or endocannabinoid system modulation. In this study, we investigated whole cannabis flower extracts, rich in diverse active compounds, for their ability to inhibit key AD-related enzymes: acetylcholinesterase (AChE), butyrylcholinesterase (BChE), and β-secretase (BACE1), using spectrophotometric assays. Fractionation of one extract further identified components with potent inhibitory activity against both BChE and BACE1.

Additionally, the extracts were evaluated for their cell-protective potential in PC12 cells exposed to hydrogen peroxide (H₂O₂) and amyloid β (Aβ₁–₄₂), mimicking AD-like oxidative stress conditions. Cannabis extracts showed stronger inhibition of cholinergic enzymes compared to Galantamine, with efficacy depending on extract composition. Importantly, this study is among the first to reveal significant BACE1 inhibition by cannabis extracts. Moreover, the extracts enhanced PC12 cell viability under oxidative stress, indicating cell-protective effects.

Overall, these findings suggest that multi-component cannabis extracts may interact with several AD-related molecular targets in vitro and may serve as promising leads for further mechanistic and preclinical investigation.

**Supplementary Information:**

The online version contains supplementary material available at https://doi.org/10.1186/s42238-026-00479-9.

## Introduction

Alzheimer's disease (AD) is a chronic neurodegenerative disease characterized by a progressive decline in cognitive function, memory loss, and dementia (Alzheimer’s Association 2022). AD is the most common chronic neurodegenerative disease worldwide (Peng et al. 2023), and the fifth leading cause of death in the adult population, For example, 6.5 million Americans over 65 lived with AD and other dementia-related illnesses, which caused 121,499 deaths in 2019 ^1^, (Yao et al. 2023). The number of patients with AD and other cause dementia worldwide is expected to exceed 150 million by 2050, according to the 2022 Alzheimer’s Disease Facts and Figures report (Alzheimer’s Association 2022). Despite extensive research, the etiology of AD is complex, and its pathogenesis remains unknown. However, various factors are contemplated in the pathophysiology of the disease, such as, neuroinflammation/microglial activation and cerebrovascular, the formation of amyloid β-protein (Aβ) plaques, low levels of acetylcholine, oxidative stress, abnormal posttranslational modifications of tau protein, hyperphosphorylation, truncation, and acetylation (Tumiatti et al. 2010; Faraji et al. 2019). Aβ peptides, central to the amyloid cascade hypothesis, are derived from the Amyloid Precursor Protein (APP) through sequential cleavage by β-secretase (BACE1) and γ-secretase. Thus, inhibition of BACE1 has considered a promising therapeutic approach in preventing AD (Zhang et al. 2019; Cai et al. 2001; Cole and Vassar 2007). Two enzymes, acetylcholinesterase (AChE) and butyrylcholinesterase (BChE), are involved in the hydrolysis of acetylcholine, a neurotransmitter that plays an important role in the process of learning and memory in the hippocampus, and lowering its level during the development of AD. Therefore, the inhibition of AChE and BChE is a highly desirable strategy for the treatment of AD (Jannat et al. 2019; Chaudhary et al. 2018; Balducci and Forloni 2018). Currently, the FDA-approved drugs for AD are AChE inhibitors, such as, donepezil, galantamine, and rivastigmine, which improve short-term memory and cognitive levels in addition to memantine (NMDA receptor antagonist) and anti-amyloid mAbs (e.g., lecanemab; region-specific approvals). However, the wide range of side effects associated with these drugs, such as nausea, vomiting, diarrhea, cardiac arrhythmias, and their failure to modify disease progression, has strengthened researchers’ motivation to explore novel treatments for Alzheimer’s disease (Ahmad et al. 2015; Ayaz et al. 2015; Sharma 2019).

Several cells characterized by neuronal properties are widely used to mimic the pathological process of AD, such as PC12, SH-SY5Y, and N2a, especially the PC12 pheochromocytoma cell line. PC12 cells are a rat adrenal pheochromocytoma cell line, a clonal cell line transplanted from rat adrenal pheochromocytoma (tumor of the adrenal medulla) by Greene and Tischler in 1976 (Greene and Tischler 1976). Although PC12 are rat tumor cells, not human neurons, these cells were widely used for the screening of natural compounds that could prevent neuron cells from cytotoxicity induced by H_2_O_2_ and Aβ (Wang et al. 2018; Xie et al. 2023). PC12 are considered as sympathetic neuron-like cells, and they are sensitive to apoptosis inducers such as H_2_O_2_ and Aβ. In addition, the Aβ oligomer combined with an integrator simultaneously induces the excitement of tyrosine kinase dependence on the N-methyl-D-aspartate (NMDA) receptor which is expressed by PC12 cells (Xie et al. 2023). PC12 cells, generally do not express endogenous CB1 or CB2 receptors, making them useful for studying cannabinoid effects independent of native cannabinoid signaling.

Cannabis is a natural plant with a long history of human use, particularly in therapeutics. The Cannabis plant and its products consist of an enormous variety of chemicals from several natural product classes, mainly Cannabinoids and Terpenes (Flores-Sanchez and Verpoorte 2008; Mahlberg and Kim 2004). The potential of terpenes as anti-AD agents were previously reported (Lima and Medeiros 2024). Cannabinoids are unique phytochemicals found only in this plant. More than 100 cannabinoids are known; all are biosynthesized from cannabigerolic acid (CBGA) or cannabigerivarinic acid (CBGVA) as carboxylic acids and can be found in the plant usually with the carboxyl group, which is decarboxylated (loss of CO2) in the presence of heat or light (Kostanda and Khatib 2022). The therapeutic value of those compounds is enormous, presenting, among others, anti-inflammatory, antioxidant, neuroprotective, and anti-pain properties. Given the complexity of AD, where single-target therapies often prove inadequate, a multi-target approach utilizing cannabis-derived compounds may offer promising alternatives (Bahji et al. 2022; Swenson 2025; Doumar et al. 2025; Reis et al. 2018). In the present study, we examined the in vitro potential of cannabis extracts to modulate AD-related molecular targets. Cannabis inflorescences from different chemovars were extracted, and their inhibitory effects on AChE, BChE and BACE1 enzymes were examined. The ability of the cannabis extracts in preventing PC12 cells cytotoxicity induced by H_2_O_2_ and Aβ1–42 oligomers was also explored.

## Materials and methods

### Materials

Human Acetylcholinesterase (AChE; EC 3.1.1.7) (Cat#. C1682), Human Butyrylcholinesterase (BChE; EC 3.1.1.8) (Cat#. B4186), Acetylthiocholine Iodide (ATCI) (Cat#. 01480), Butyrylthiocholine iodide (BTCI) (Cat#. B3253), 5,5-dithio-bis-nitrobenzoic acid (DTNB) (Cat#. D8130), Galantamine Hydrobromide (Cat#. PHR1623), Collagen (Cat#. C9791), 3- (Peng et al. 2023; Tumiatti et al. 2010; Faraji et al. 2019)−2,5-diphenyl-tetrazolium bromide (MTT) (Cat#. 475,989), Potassium phosphate buffer (pH 8.0) and dimethyl sulfoxide (DMSO) obtained from Sigma-Aldrich. CellTiter 96® AQueous One Solution Cell Proliferation Assay (MTS) obtained from Promega. β-site amyloid precursor protein cleaving enzyme 1 (BACE 1; EC 3.4.23.46) Inhibitor Screening Kit (Cat#. AB283408) and Human beta amyloid 1–42 (Aβ1–42) peptide (synthetic, Cat#. AB120301) obtained from Abcam. PC12 cells CRL-1721obtaine from ATCC. RPMI 1640 medium, heat-inactivated horse serum, fetal bovine serum, and Penicillin–Streptomycin Solution obtained from Sartorius.

### Plant material, extraction, and fractionation

*Cannabis sativa L.* plant inflorescences were obtained from Prof. Moshe Fleishman (Bar Lev Institute, Israel). The inflorescences were crushed in a laboratory mortar and pestle with the addition of liquid nitrogen. The crushed inflorescences underwent a process of partially decarboxylation in a laboratory oven at a temperature of 100° C for 1 h. Inflorescence powder was extracted with 100% ethanol at a 1:20 ratio for 1 h at room temperature and filtered. The residual plant material was re-extracted with 50% of the initial ethanol volume for 30 min and filtered. The combined filtrates were evaporated using a Rotavapor® R-100 (BUCHI, Flawil, Switzerland). The extracts were stored in a freezer at −20°C until use. For fractionation, 100 mg of the crude ethanolic extract C3 was dissolved in dichloromethane, mixed with Celite and subject to Pure C-815 Flash system (BUCHI). fractionation was performed on BUCHI FP ECOFLEX silica cartridge (4g, lot. 140,000,021), separation was carried out using (A) Hexane, (B) Dichloromethane, (C) Ethyl acetate, and (D) Methanol as the mobile phases. The solvent gradient was adjusted during the run according to chromatographic profiles, at a flow rate of 15 mL/min. UV absorbance was monitored simultaneously at 254–365 nm. Forty fractions were collected based on time/volume; fractions sharing similar TLC characteristics were pooled to yield ten combined fractions, which were evaporated under reduced pressure and dried under nitrogen. Subsequent analytical evaluation resulted in six final pooled fractions. Dried extracts and fractions were reconstituted in ethanol or DMSO (w/v) and subsequently diluted in assay buffer or culture medium. The final solvent concentration in all bioassays did not exceed 1% (v/v).

### Analytical characterization by LC–MS/MS

The cannabinoid analysis was performed using a method previously developed in our laboratory by E. Kostanda (Kostanda and Khatib 2022). In brief, we utilized a Q Exactive™ Plus Quadrupole-Orbitrap Mass Spectrometer (Thermo Fisher Scientific) coupled to a Dionex Ultimate 3000 UHPLC system, operating with a HESI-II (Heated Electrospray Ionization) source. For each compound, a calibration curve was constructed using a cannabinoid standard mix at concentrations ranging from 0.019 to 10 ppm, with an internal standard (IS), CBD-D3, at a concentration of 0.1 ppm. The calibration curve was run under the same conditions as the sample analyses. Extract samples at a concentration of 10 ppm, diluted in methanol and containing the IS, were injected (5 μL) into a Poroshell 120 EC-C18 column (3.0 × 150 mm, 2.7 μm; Agilent Technologies). The mobile phase consisted of (A) double-distilled water (DDW) with 0.1% formic acid and (B) acetonitrile with 0.1% formic acid. Separation was achieved using the following mobile phase gradient: 0–3 min, 60% B; 3–7 min, 60%–80% B; 7–12 min, 80%–90% B; 12–20 min, 90% B; 20–22 min, 90%–60% B; and 22–25 min, 60% B hold. The flow rate was 0.4 mL/min. The column temperature was maintained at 30 °C, and the autosampler tray was held at 10 °C. Cannabinoids were detected in positive ionization mode, with quantification based on the [M + H]^+^ ion. The MS source temperature was set to 350 °C, and the capillary voltage was 3.5 kV. HR scan mode was used with the mass range m/z 150–800 with a resolution of 70,000. Identification was based on retention times and molecular masses of the analytes. Data were analyzed using Xcalibur and Freestyle software.

### Cholinesterase inhibition assay

The Acetylcholinesterase and Butyrylcholinesterase inhibitory properties of the Cannabis extracts and fractions were evaluated using a spectrophotometric method previously developed by Ellman et al. (Ellman et al. 1961). Human AChE and Human BChE were used, while acetylthiocholine iodide and butyrylthiocholine iodide, respectively, were used as substrates. 5,5′-Dithio-bis (Peng et al. 2023) (DTNB) was used for the measurement of the cholinesterase activity. Briefly, to a 96-well, clear, F-bottom plate, 120 μL of PBS buffer (pH 8.0), 20 μL of test samples (at final concentration ranging from 0.5 to 2.5 μg/mL), and 20 μL of AChE (0.25 U/mL) or BChE (fold diluted-70) were added. The plates were incubated in the dark at room temperature for 30 min or at 37 °C for 20 min, respectively. Next, 20 μL of 1 mM DTNB and 20 μL of 7.5 mM ATCI or 4 mM BTCI were added to the reaction mixture and were analyzed at 412 nm. Absorption was recorded kinetically for 20 min. Enzyme activity was calculated from the initial linear rate of reaction (ΔA/min). Measurements were performed in technical triplicates. Background correction was performed using blank wells containing all assay component except enzyme. Galantamine, a known AChE inhibitor, served as the positive control. Inhibition percentages were calculated as described before (Ahmad et al. 2015).

### BACE1 inhibition

BACE1 enzyme inhibition was measured using the commercial BACE1 Inhibitor Screening Kit (Abcam, Cat# ab283408) according to the manufacturer's protocol. Cannabis extracts at a final concentration of 10, 20, and 50 μg/mL were added to a white 96-well plate (Greiner) with buffer and enzyme, and the plate was incubated for 5 min at room temperature. Then, the substrate was added, and the plate was immediately read in a plate reader (BioTek Synergy H1). Absorbance was measured at 345 nm, and emission at 500 nm for 60 min. For each analysis, a negative control was performed in which no enzyme was added, and a positive control was performed in which no inhibitor was added. In order to assess the overall BACE1 inhibition, the area under the curve (AUC) was calculated for each curve in the kinetic assay (Savva et al. 2023).

### PC12 cell culture treatment

PC12 cells were purchased from ATCC (USA). Cells were cultured in RPMI 1640 medium supplemented with 10% heat-inactivated horse serum, 5% fetal bovine serum, and 1% Penicillin–Streptomycin Solution at 37 °C in a humidified atmosphere of 5% CO_2_. Cells were subcultured every 2–3 days. The experiments were performed on cells between passages 15 and 25. For the viability assay, cells were seeded at a density of 1 × 10^5^ cells/well in 96-well culture Collagen-coated plates (Wiatrak et al. 2020). After plating, the cells were left for 24 h for adhesion to the surface of the culture vessel.

### Cell viability using MTT Assay

MTT assay was used to determine cellular mitochondrial dehydrogenase activity. Briefly, 24 h after being cultured in a 96-well collagen-coated plate, the PC12 cells were treated with Cannabis extracts or fractions at different concentrations together with 1μM amyloid beta oligomers (Stine et al. 2011) for another 24 h. After incubation, cells were treated with the MTT solution (final concentration 0.5 mg/mL) for 3 h. The dark blue formazan crystals formed in intact cells were solubilized with DMSO, and absorbance at 550 nm was measured with a microplate reader.

### Cell viability using MTS assay

The CellTiter 96® AQueous One Solution Cell Proliferation Assay (Promega, Cat# PR-G3582) was employed according to the manufacturer's instructions. Briefly, PC12 cells (1 × 104 cells/mL) were seeded in 96-well plates at 100 µl/well and incubated for 4 h. After being cultured in a 96-well plate at a density of 10 × 104 cells/mL. Following initial incubation, cannabis extract was added to the wells. After 24 h of incubation, the supernatant was discarded, and a 300 μM hydrogen peroxide solution was added to each well for an additional 24 h. After incubation, MTS reagent was added to each well, and after 3 h, the absorbance of the plate was measured at 490 nm.

### Statistical analysis

The statistical tests were performed using Prism 9 software (GraphPad Software, CA, U.S). Data are expressed as mean ± SEM. Statistical analysis was performed using an unpaired t-test or one-way parametric ANOVA, depending on the experimental structure. The concentration ranges used in the AChE, BChE, BACE1, and PC12 cell viability assays were selected based on preliminary optimization experiments performed in our laboratory. Because each assay differs in enzymatic kinetics, sensitivity, detection method, and biological tolerance to the extracts or fractions, assay-specific concentration ranges were used to obtain measurable, non-saturated responses and to avoid cytotoxic concentrations in cell-based experiments.

In order to calculate the IC_50_ values, non-linear regression tests were performed—[inhibitor] vs. normalized response -Variable slope.

## Results

### Cannabis Inflorescence extraction and cannabinoids profile characterization:

Inflorescences from four chemovars of *Cannabis sativa L.* were heated to 100 °C, extracted using 100% ethanol, and their cannabinoid profiles were characterized using the LC-MS/MS method previously established within our laboratory (Kostanda and Khatib 2022). 14 common cannabinoids were analyzed and quantified across the four selected extracts, and the results are detailed in Table 1. The heating step achieved partial decarboxylation, as reflected by the CBD/CBDA and THC/THCA ratios derived from the LC-MS/MS profiles. All the extracts exhibited high levels of CBD; In contrast, extracts C1, C3, and C4 were classified as Type II chemovars (contain both THC and CBD) based on Δ9-THC:CBD ratios of approximately 0.8–1.3, whereas extract C2 was classified as a Type III chemovar (CBD-predominant) due to a markedly lower Δ9-THC:CBD ratio (~0.05), consistent with established chemotype definitions (Lewis et al. 2018). The content of other cannabinoids varies among the extracts. In particular, extract C2 significantly differs (p<0.05) from the other three extracts in the concentrations of CBD, THC, and CBC. In addition, extracts C3 and C4 contain CBGA and CBNA, which are not detected in extracts C1 and C2 (Table 1).

**Table 1:** Quantitative LC–MS/MS Cannabinoids profile of four *Cannabis sativa L.* extracts (C1-C4). Values are expressed as mg/g dry extract ± SEM. (*n* = 4), ND = Not detected

Cannabinoid (mg/g)	**C1**	**C2**	**C3**	**C4**
CBDV	ND	ND	ND	ND
CBDA	174.4 ± 12.6	261.4 ± 19.7	226.6 ± 14.7	205.2 ± 17.5
CBGA	ND	ND	7.3 ± 1.3	3.9 ± 1.1
CBG	1.7 ± 0.6	2.6 ± 0.5	4.4 ± 0.5	2.9 ± 0.6
CBD	185.5 ± 8.0	277.4 ± 25.1*	117.4 ± 5.8	141.7 ± 10.4
THCV	ND	ND	ND	ND
CBN	2.6 ± 0.2	ND	7.4 ± 0.6	11.9 ± 1.4
Δ⁹-THC	155.7 ± 8.3	14.03 ± 1.8*	148.9 ± 8.8	163.2 ± 14.4
CBNA	ND	ND	2.9 ± 0.9	3.3 ± 1.5
Δ⁸-THC	ND	ND	ND	ND
CBL	ND	ND	ND	ND
CBC	7.6 ± 0.9	12.2 ± 0.8*	5.1 ± 0.5	6.6 ± 0.5
THCA	38.7 ± 3.1	ND	57.1 ± 4.0	51.9 ± 4.5
CBCA	3.9 ± 1.2	6.7 ± 1.1	8.0 ± 1.4	7.6 ± 1.4

*Abbreviations:CBDV* Cannabidivarin, *CBDA* Cannabidiolic acid, *CBGA* Cannabigerolic acid, *CBG* Cannabigerol, *CBD* Cannabidiol, *THCV* Tetrahydrocannabivarin, *CBN* Cannabinol, Δ⁹-*THC* Delta-9-tetrahydrocannabinol, *CBNA* Cannabinolic acid, Δ⁸-*THC *Delta-8-tetrahydrocannabinol, *CBL* Cannabicyclol, *CBC* Cannabichromene, *THCA* Tetrahydrocannabinolic acid, *CBCA* Cannabichromenic acid. Statistical tests (one-way ANOVA with Tukey’s post-hoc) were conducted for each cannabinoid. Cannabinoids showing significantly different concentration in one extract compared to the other three are indicated with an asterisk (*)

## The effect of the extracts on the activity of the enzymes AChE, BChE, and BACE1

### The effect of the extracts on AChE activity

**Fig. 1:**
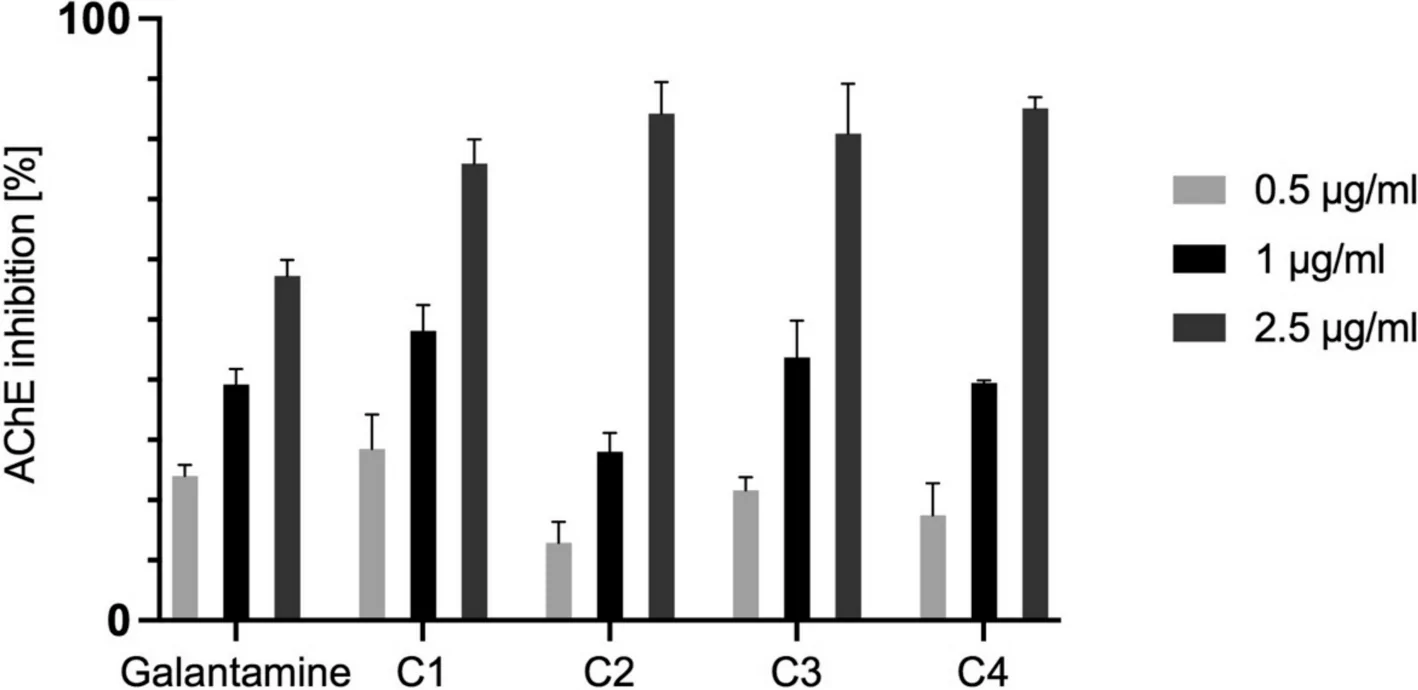
AChE inhibition. The inhibition capacity of the extracts and galantamine on AChE was evaluated at three different concentrations using spectrophotometric Ellman assay. The high concentration of galantamine used in a concentration of 2 µg/mL. Results are presented as mean ± SEM (*n* = 3)

The effects of each extract on the activity of the AChE enzyme were examined using the spectrophotometric Ellman assay (Ellman et al. 1961). All the extracts demonstrated enzyme inhibition in a dose-dependent manner. When compared with galantamine, the extracts exhibited comparable levels of inhibition, with no significant differences observed between the extracts (Fig. 1).

### The effect of the extracts on BChE activity

**Fig. 2:**
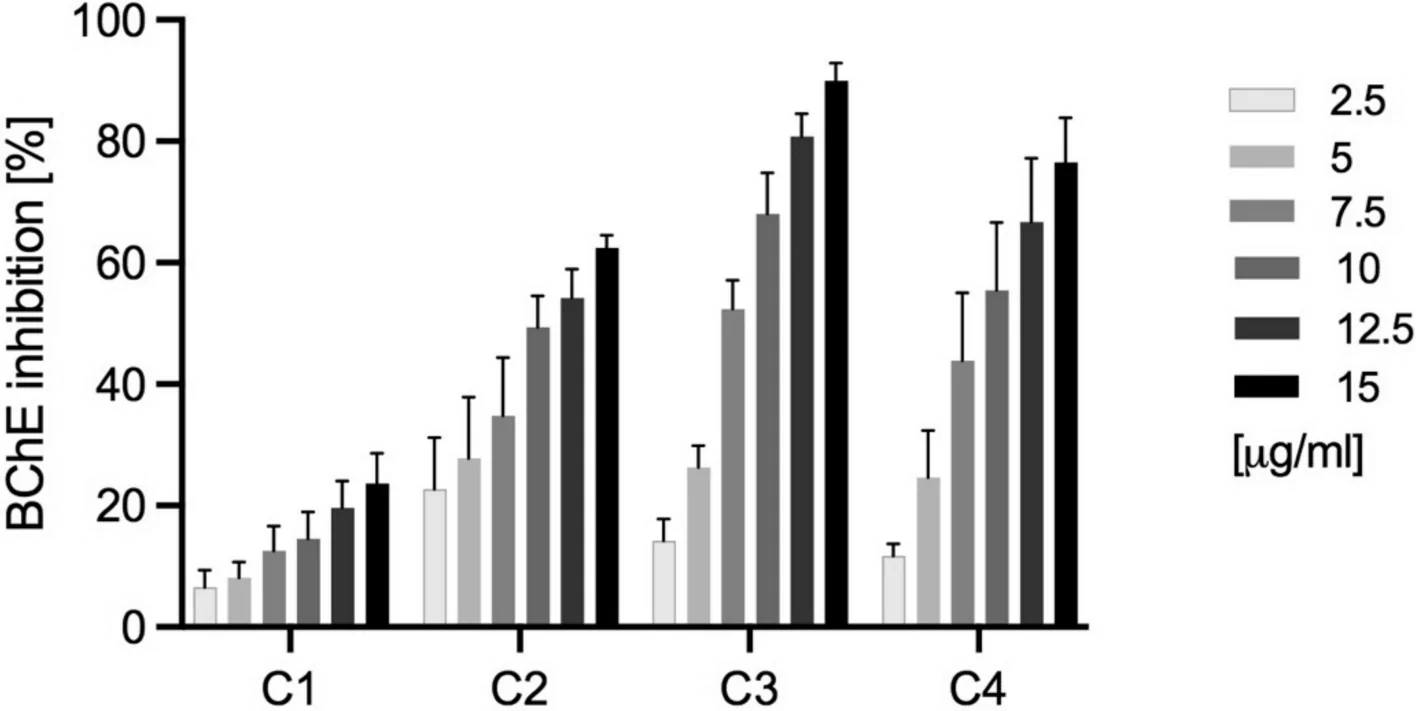
BChE inhibition. Inhibition percentage profile of BChE activity by extracts at varying concentrations 2.5, 5, 7.5, 10, 12.5, 15 μg/mL. Results are presented as mean ± SEM (*n* = 3)

The effects of each extract on the activity of the BChE enzyme were examined using the spectrophotometric Ellman assay. All extracts inhibited the activities of BChE enzymes in a concentration-dependent manner (Fig. 2). The inhibition IC_50_ values of extracts C2, C3, and C4 were 9.9, 7.19, and 9.06 µg/mL, respectively; all of them inhibited the enzyme with IC_50_ approximately four times lower than that of Galantamine (35.93 µg/mL) (Table 2). However, C1 inhibited the enzyme with an IC_50_ of 35.81 µg/mL.

**Table 2:** BChE IC_50_ values of the extracts and Galantamine. Statistical analysis was performed using one-way ANOVA followed by Dunnett’s multiple comparisons test versus Galantamine. Values (µg/mL) are presented as mean ± SEM. Each group included three biological replicates, except for C1 (*n* = 2). *p < 0.005

Extracts	IC_50_ (mean ± SEM)	p vs Galantamine
C1	35.81 ± 13.7	ns
C2	9.90 ± 3.8	***
C3	7.19 ± 1.0	***
C4	9.06 ± 2.9	***
Galantamine	35.93 ± 2.5	-

### The effect of the extracts on BACE1 activity

**Fig. 3:**
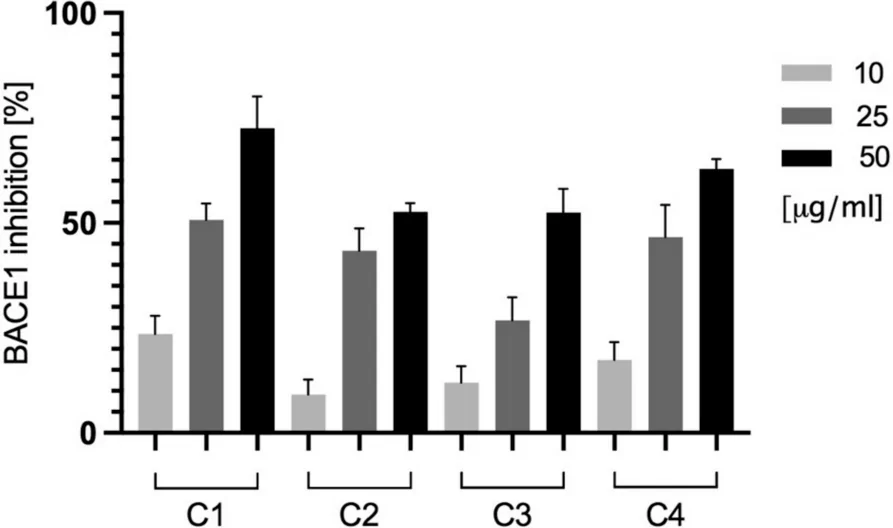
BACE1 inhibition. Inhibition percentage profile of BACE1 activity by extracts at the concentrations 10, 25, 50 μg/mL. Results are presented as mean ± SEM (*n* = 3)

The effects of each extract on the activity of the BACE1 enzyme were examined using the commercial BACE1 Inhibitor Screening Kit (Abcam, Cambridge, UK). All four extracts demonstrated the ability to inhibit BACE1 enzyme activity in a concentration-dependent manner (Fig. 3). At a concentration of 10 μg/mL, the inhibition values ranged from 7.9% to 21.3%. At 25 μg/mL, the inhibition values ranged from 26.6% to 51%. At 50 μg/mL, the inhibition values ranged from 47.9% to 65.3%. No statistically significant differences were observed between the extracts at the same concentration. inhibition values were derived from the area under the fluorescens time curve. Estimated IC₅₀ values for BACE1 inhibition, calculated from the available three-point dose–response data, were 24.3 μg/mL, 40.2 μg/mL, 47.8 μg/mL, and 30.8 μg/mL for extracts C1, C2, C3, and C4 respectively. The estimated BACE1 IC₅₀ values were calculated from only three concentration points and should therefore be interpreted with caution.

## The cell-protective properties of the extracts using PC12 cells

### The ability of the extract C3 to protect PC12 cells from toxicity induced by amyloid beta (Aβ_1–42_):

Although several extracts showed comparable inhibitory activity in individual assays, extract C3 was selected for further cellular evaluation and fractionation because it demonstrated the most integrated pharmacological and biological profile across the evaluated assays, together with a favorable phytochemical composition. Specifically, C3 exhibited the lowest BChE IC₅₀ among the crude extracts, clear inhibitory activity against AChE and BACE1, and a cannabinoid profile comprising both neutral and acidic cannabinoids

**Fig. 4:**
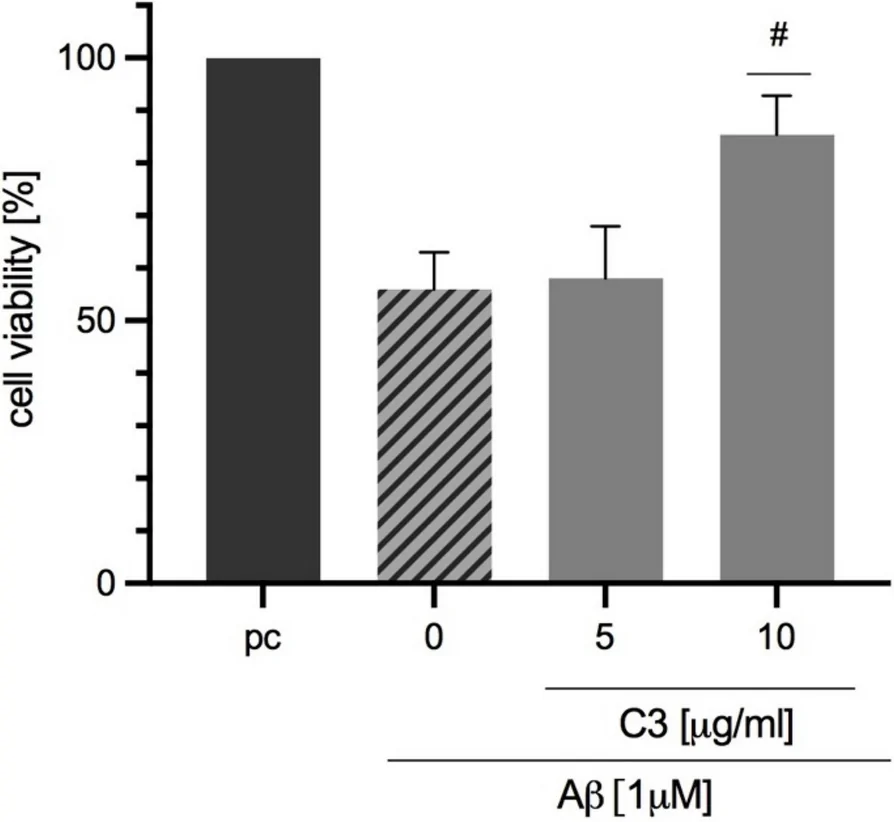
Cell-protective effects of extract C3 on PC12 cells. increasing concentrations of the C3 extract enhance cell viability compared to the 0 µg/mL group, with the optimal concentration being 10 µg/mL. Statistical analysis was performed using an unpaired t-test comparing each treatment group to the 0 µg/mL group. Results are presented as mean ± SEM. *p < 0.05, (*n* = 5)

The ability of extract C3 to protect PC12 cells from toxicity induced by amyloid beta oligomers was investigated. Based on preliminary tests, amyloid beta was used at a concentration of 1 µM. Results from the MTT cell viability assay and the calculation of cell viability percentages relative to the positive control (PC=untreated cells) indicated that increasing concentrations of the extract corresponded to higher cell viability compared to untreated cells. Optimal protection was observed at a concentration of 10 µg/mL (Fig. 4).

### The ability of extract C3 to protect PC12 cells from toxicity induced by Hydrogen peroxide (H_2_O_2_)

**Fig. 5:**
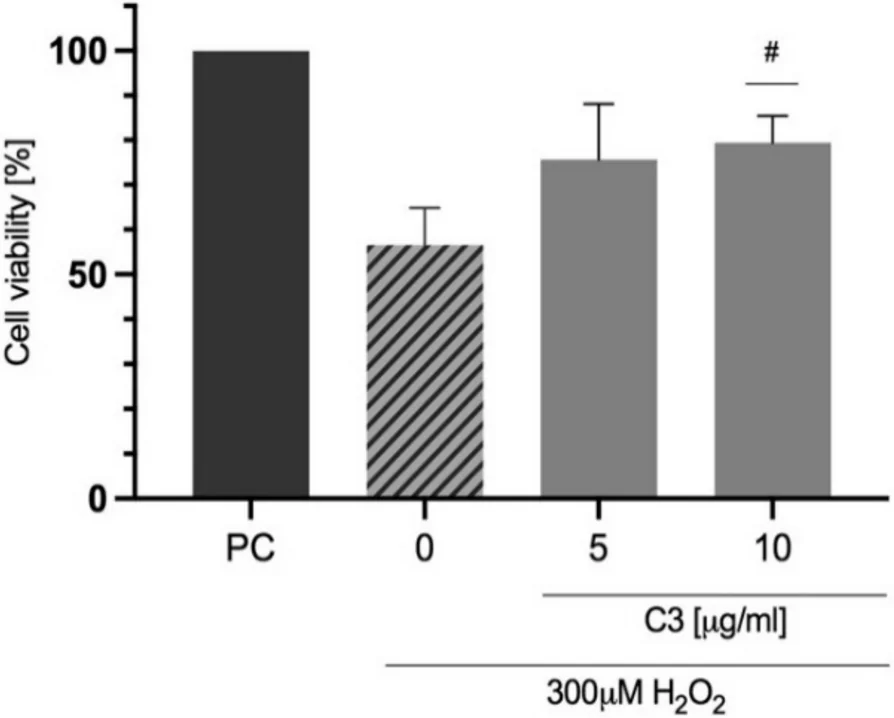
Cell-protective effects of extract C3 on PC12 cells. increasing concentrations of the C3 extract enhance cell viability relative to untreated cells, with the optimal concentration being 10 µg/mL. Statistical analysis was performed using an unpaired t-test comparing each treatment group to the 0 µg/mL group. Results are presented as mean ± SEM. #*p* < 0.05, (*n* = 3)

The ability of the extracts to protect PC12 cells from oxidative stress induced by hydrogen peroxide was assessed. Following preliminary tests, extract C3 was applied at a concentration of 5 and 10 µg/mL, and H_2_O_2_ was used at 300 µM. Results from the MTS cell viability assay indicated that pretreatment with C3 increased the cell viability compared to untreated cells in a dose-dependent manner. At a concentration of 10 µg/mL of extract, the increase in cell viability was statistically significant (Fig. 5).

## Fractionation of the extract C3

Extract C3 was separated into fractions using direct phase chromatography to obtain six fractions, f1-f6, which were analyzed using LC–MS/MS for their cannabinoid content (Table 3). Fractions f1 and f2 contain a higher concentration of the neutral cannabinoids THC and CBD; f3 and f4 contain a higher concentration of the cannabinoid CBDA. Fractions f5 and f6 contain the cannabinoids CBGA in addition to CBDA.

**Table 3:** LC–MS/MS Cannabinoids profile of six fractions obtained from extract C3. (mg/g) ± SEM. (n = 3), ND = Not detected

Cannabinoid (mg/g)	**f1**	**f2**	**f3**	**f4**	**f5**	**f6**
CBDV	ND	6.2 ± 0.09	ND	ND	ND	ND
CBDA	ND	164.9 ± 11.9	552.3 ± 19.5	339.6 ± 3.8	155.6 ± 3.6	17.67 ± 0.93
CBGA	ND	ND	ND	1.3 ± 0.2	81.3 ± 3.9	7.8 ± 0.2
CBG	ND	ND	3.6 ± 0.5	29.6 ± 3.8	ND	ND
CBD	329.4 ± 13.9	242.5 ± 8.7	15.1 ± 2.2	8.7 ± 1.4	2.2 ± 0.3	ND
THCV	6.7 ± 0.4	2.9 ± 0.1	ND	ND	ND	ND
CBN	70.3 ± 3.9	54.6 ± 3.3	11.11 ± 1.7	ND	ND	ND
Δ⁹-THC	440.5 ± 12.1	84.4 ± 3.7	3.9 ± 0.7	ND	ND	ND
CBNA	ND	58.9 ± 4.2	40.1 ± 7.7	10.3 ± 1.2	12.9 ± 0.8	31.0 ± 2.8
Δ⁸-THC	ND	ND	ND	ND	ND	ND
CBL	ND	ND	ND	ND	ND	ND
CBC	ND	ND	ND	ND	ND	ND
THCA	ND	172.0 ± 62.6	57.4 ± 55.0	ND	ND	ND
CBCA	ND	ND	190.0 ± 27.8	ND	ND	ND

## The effect of the fractions on the enzymes AChE, BChE, and BACE1

### The effect of the extracts on AChE activity

**Fig. 6:**
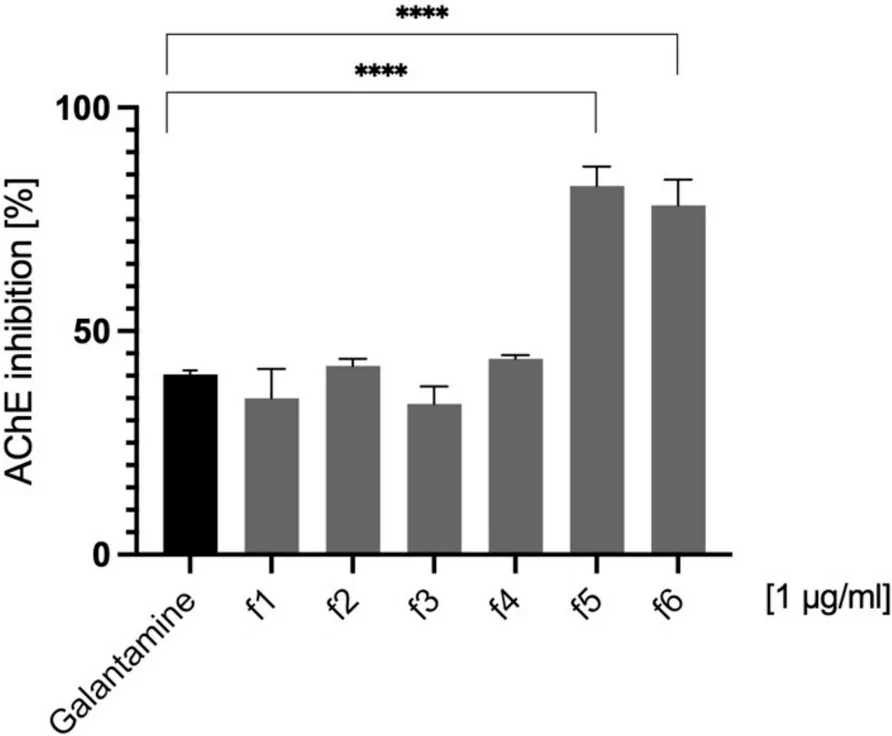
AChE inhibition. The impact of the fractions on AChE activity was assessed in comparison to galantamine. Fractions f5 and f6 exhibited the most substantial reduction in enzyme activity. Results are presented as mean ± SEM (*n* = 3)

The inhibitory effects of the fractions on AChE were assessed. At a concentration of 1 µg/mL, fractions f1-f4 inhibited the AChE activity by 40% similar to Galantamine. In contrast, fractions f5 and f6 inhibited the enzyme activity by 85% at the same concentration, which was significantly higher than Galantamine (Fig. 6).

### The effect of the fractions on BChE activity:

**Fig. 7:**
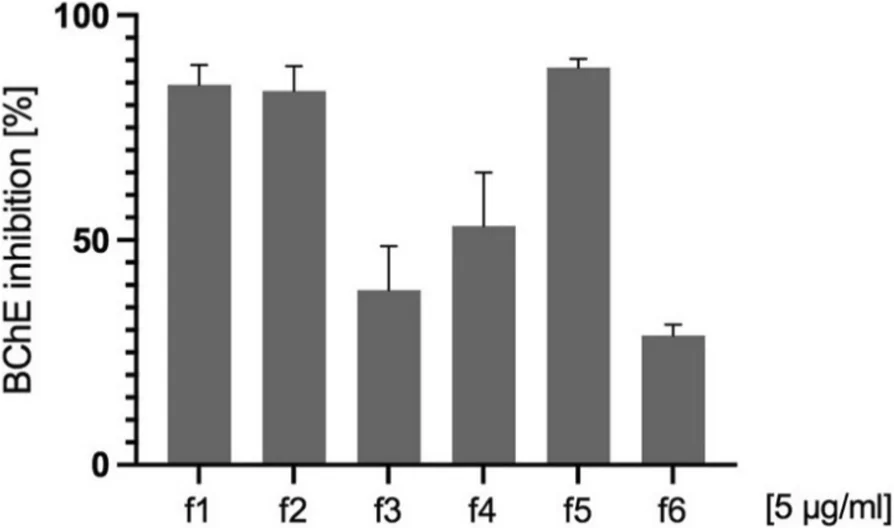
BChE inhibition by fractions. f1, f2, and f5 exhibited the most substantial inhibitory activity. Results are presented as mean ± SEM (*n* = 3)

The inhibitory effect of the fractions on BChE was subsequently evaluated. All the fractions at a concentration of (5 μg/mL) inhibited the enzyme activity by a different percentage, as shown in Fig. 7. Fractions f1, f2, and f5 inhibited the enzyme by 80%, while the other fractions inhibited it by 30–50%. The IC_50_ of inhibition was evaluated for the most active fractions: f1 (1.4 µg/mL), f2 (3.5 µg/mL), and f5 (2.5 µg/mL). The IC_50_ values of the fractions were significantly lower than those of the crude extract C3 (7.2 µg/mL). In addition, f1 and f5 were at least 50% lower than C3 (Table 4).

**Table 4:** BChE IC_50_ values of chosen fractions and C3. Statistical analysis was performed using one-way ANOVA followed by Dunnett’s multiple comparisons tes, with C3 as the reference group. values (µg/mL) are presented as mean ± SEM. (*n* = 3) ***p < 0.001, ****p < 0.0001

	IC_50_ (mean ± SEM)	p vs C3
f1	1.47 ± 0.3	****
f2	3.56 ± 0.36	***
f5	2.5 ± 0.6	****
C3 (Crude)	7.19 ± 1.09	-

### The effect of the fractions on BACE1 activity:

**Fig. 8:**
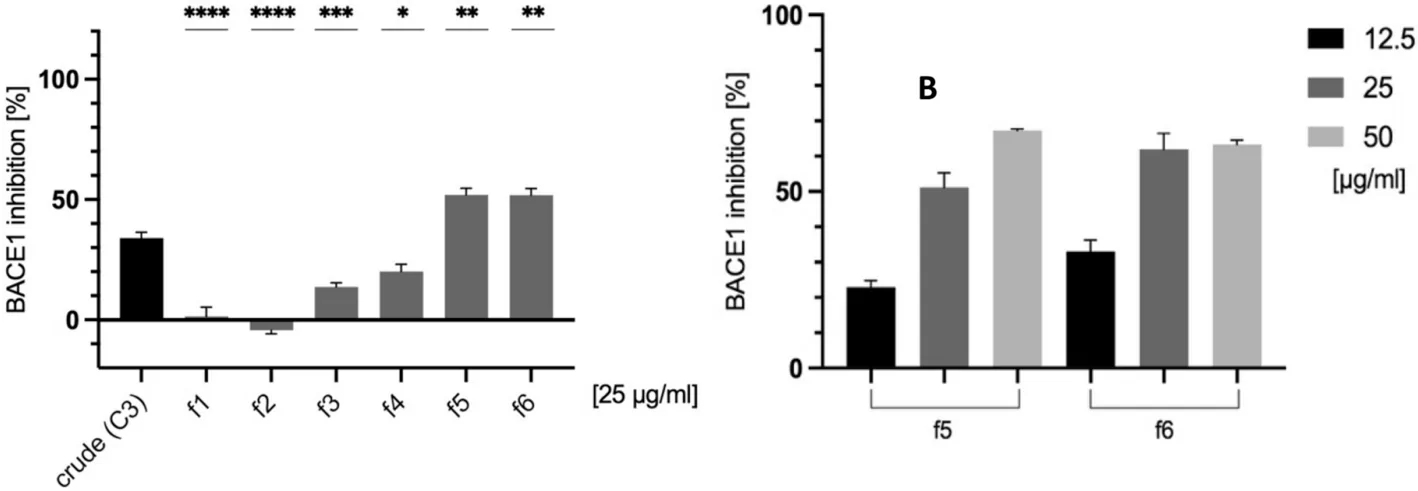
BACE1 inhibition. **A** The effect of the fractions on enzyme activity was compared to that of the crude C3 extract at same concentration. **B** The effect of fractions f5 and f6 on enzyme activity using different concentrations. Results are presented as mean ± SEM (*n* = 3). * < 0.05, ** < 0.01, *** < 0.001, **** < 0.0001

Upon evaluating the inhibition capacities of the fractions on BACE1 activity it was found that only two fractions, f5 and f6 inhibited the enzyme by 50% percent at a concentration of 25 μg/mL, and significantly higher than the crude extract C3 (Fig. 8A). The inhibition ability of fractions f5 and f6 on BACE1 were increased at a dose dependent manner (Fig. 8B). Estimated IC₅₀ values for BACE1 inhibition, were 27.2 μg/mL, and 21.7 μg/mL for fractions f5, and f6 respectively. The estimated BACE1 IC₅₀ values were calculated from only three concentration points and should therefore be interpreted with caution.

### The ability of f5 to protect PC12 cells from toxicity induced by amyloid beta (Aβ_1–42_)

**Fig. 9:**
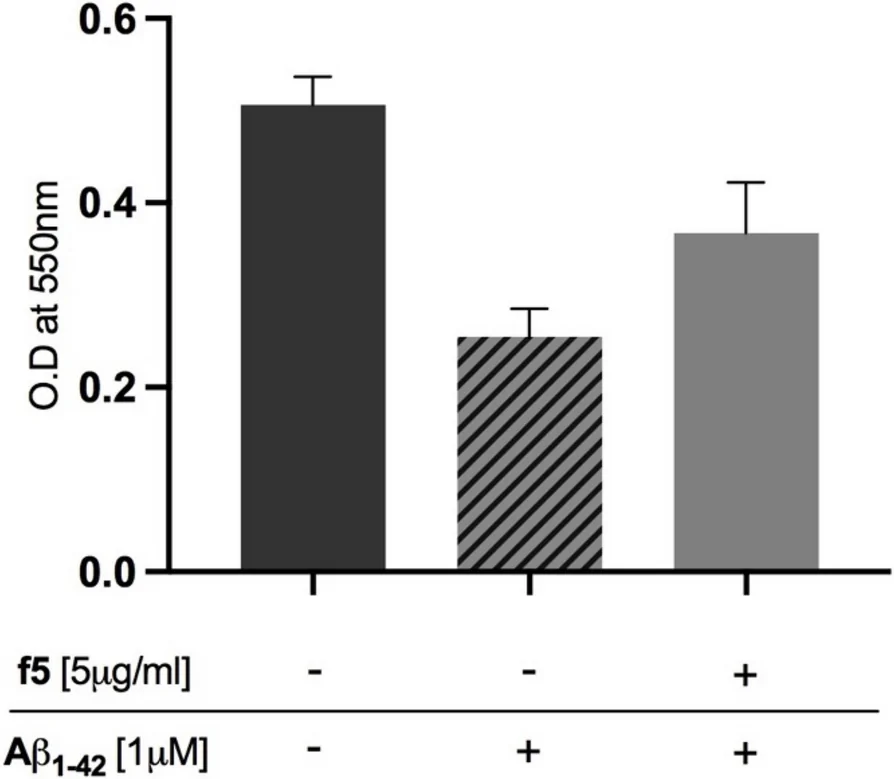
Cell-protective effects of fraction f5 on PC12 cells. f5 fraction at a concentration of 5µg/mL increased cell viability relative to cells treated just with Aβ_1–42_ (The increase was statistically non significance). Results are presented as mean ± SEM (*n* = 3)

The ability of fraction f5 to protect PC12 cells from toxicity induced by amyloid beta oligomers was investigated. Based on preliminary tests, amyloid beta was used at a concentration of 1 µM. Results from the MTT cell viability assay showed that f5 at 5 µg/mL increased PC12 cell viability compared with cells treated with Aβ₁–₄₂ alone; however, this increase was not statistically significant (Fig. 9).

## Discussion

Alzheimer’s disease, a condition characterized by multifactorial pathogenesis involving cholinergic deficits, β-amyloid aggregation, tau hyperphosphorylation, and oxidative stress (Raïch et al. 2025; Marsh et al. 2024). The present study demonstrates that cannabis-derived extracts can modulate several Alzheimer’s disease-related targets in vitro, our findings reveal that ethanol extracts from different *Cannabis sativa L.* chemovars, as well as their chromatographic fractions, exhibited inhibitory activity against acetylcholinesterase (AChE), butyrylcholinesterase (BChE), and β-site amyloid precursor protein cleaving enzyme 1 (BACE1).

Among the four cannabis extracts analyzed, each corresponding to a distinct chemovar (C1–C4), clear differences in phytochemical composition and biological activity were observed. LC–MS/MS analysis revealed that extracts C1, C3, and C4 were Type II chemovars containing THC and CBD, whereas C2 was a Type III chemovar dominated by CBD. Extract C3 and C4 were distinguished by its unique cannabinoid profile, containing both neutral and acidic cannabinoids, including CBGA and CBNA, which were absent in the extracts C1 and C2.

Inhibitors of AChE and BChE have been explored as pharmacological strategies to enhance acetylcholine availability, slow cognitive decline, and alleviate symptoms in AD patients. Current FDA-approved drugs such as donepezil, rivastigmine, and galantamine primarily act through AChE inhibition. Still, their limited efficacy and side effects highlight the need for novel dual AChE/BChE inhibitors with improved therapeutic profiles (Mesulam et al. 2002 Mesulam et al. 2002). Our results suggested that natural cannabis extracts can achieve comparable dual-cholinesterase inhibition through optimized fractionation procedures.

The cholinesterase inhibition assays (Figs. 1 and 2, and Table 2) indicate that the extracts exhibited robust AChE and BChE inhibition in vitro; some extracts exceeded galantamine’s effect at tested concentrations. This is notable given that BChE activity increases as AD progresses, making BChE a critical therapeutic target in advanced disease stages (Greig et al. 2001). Extracts C2, C3, and C4 exhibited IC₅₀ values for BChE inhibition that were significantly lower than those of galantamine, indicating notable in vitro inhibitory activity under the assay conditions used. This supports the therapeutic rationale of employing cannabis-derived phytochemicals as broad-spectrum cholinesterase inhibitors. Moreover, all four extracts inhibited BACE1 in a concentration-dependent manner (Fig. 3). Since BACE1 catalyzes the rate-limiting step in Aβ peptide generation, its inhibition represents a pivotal disease-modifying strategy (Vassar 2014). The data indicate that specific cannabinoid-enriched fractions, particularly those containing CBGA, may play a central role in this effect (Tables 1 and 2), however, this association requires confirmation by correlation analysis and testing of purified compounds. Because different concentration ranges were used across the enzymatic and cell-based assays, direct quantitative comparison between targets should be interpreted with caution. The multi-target interpretation is therefore based on the ability of the extracts and fractions to modulate several AD-related in vitro endpoints, rather than on direct potency comparison across assays.

Among the four cannabis extracts examined, extract C3 demonstrated the most promising biological activity across multiple assays. Indeed, C3 demonstrated the lowest IC₅₀ value for BChE inhibition (7.19 μg/mL) and exhibited strong AChE and BACE1 inhibition compared to the other chemovars. Furthermore, C3 provided significant cell-protection in PC12 cells against amyloid-β and oxidative stress (H₂O₂), with optimal effects observed at 10 μg/mL (Fig. 4 and Fig. 5). These combined results suggest that extract C3 contains bioactive constituents with multitarget potential relevant to Alzheimer’s disease pathology. Based on this broad spectrum of activity, C3 was selected for fractionation to isolate and identify the specific cannabinoid-enriched fractions responsible for its pharmacological effects. This approach allowed a more detailed understanding of the chemical composition of C3 and provided insights into the contribution of individual fractions and compounds to its therapeutic potential.

Our demonstration of cytoprotective effects against H₂O₂-induced cytotoxicity in PC12 cells aligns closely with recent studies on the antioxidant properties of cannabis compounds. Di Giacomo et al. (Mateos-Maces et al. 2020) demonstrated that CBD and CBG provided significant antioxidant and cytoprotective effects in rat astrocytes, with CBD showing particular efficacy against oxidative stress. Similarly, Cásedas et al. (2022) evaluated two different Cannabis sativa extracts and found substantial antioxidant activity correlated with neuroprotective effects.

Our results extend these findings by demonstrating protection in a neuronal cell model (PC12) that more closely resembles the target cells affected in AD. The consistency of cytoprotective effects across different cannabis preparations and cell models suggests that antioxidant activity is a robust and reproducible property of cannabis-derived compounds. However, although PC12 cells represent a well established neuron like model, they do not fully recapitulate the complexity of human neurons. Therefor, future validation in human iPSC derived neuronal system, including co-cultures with astrocytes or microglia, as well as functional electrophysiological readouts (e.g., LTP or multi-electrode array analyses), will be important to further align these findings with Alzheimer’s disease pathophysiology.

The mechanism by which C3 protects PC12 cells against Aβ₁-₄₂-induced toxicity is likely multifactorial. Aβ toxicity is associated with oxidative stress, mitochondrial dysfunction, membrane damage, and activation of apoptotic pathways. The ability of C3 to improve PC12 cell viability following Aβ₁-₄₂ exposure suggests that its cytoprotective activity may be mediated, at least in part, through antioxidant and cytoprotective mechanisms that preserve mitochondrial metabolic activity (Fleisher-Berkovich et al. 2024; Tong et al 2018; Park et al. 2008). This effect may also reflect additive or synergistic interactions among cannabinoids and other phytochemicals present in the extract.

Given its broad multitarget activity and rich cannabinoid diversity, extract C3 combined strong enzymatic inhibition, a broad cannabinoid profile, and sufficient available material for fractionation and downstream testing, it was selected for chromatographic fractionation to isolate and characterize the bioactive components responsible for its enhanced inhibitory and neuroprotective effects. Fractionation analysis revealed that neutral cannabinoids (CBD, THC) were abundant in f1 and f2, whereas acidic cannabinoids like CBDA and THCA predominated in fractions f3 and f4, while f5 and f6 included mainly CBDA and CBGA cannabinoids (Table 2). Interestingly, the fractions enriched in CBDA and CBGA (f5, f6) exhibited the most potent inhibition of AChE and BChE (Fig. 6 and Fig. 7), with fractions f5 and f6 exhibiting stronger inhibition for AChE activity. Fractions f1, f2, and f5 demonstrated the strongest inhibitory activity for BChE, with IC₅₀ values of 1.47, 3.56, and 2.50 μg/mL, respectively, which were all significantly lower than that of the crude extract C3 (7.19 μg/mL) (Fig. 7). These values indicate that fractionation enhanced the inhibitory capacity by concentrating the active constituents responsible for BChE inhibition. Notably, the IC₅₀ of f5 was nearly threefold lower than that of the crude extract, suggesting enrichment of highly potent cannabinoid species in this fraction. In addition, f5 at a concentration of 5 μg/mL increased cell viability compared with cells treated with Aβ₁-₄₂ alone; however, the effect was not statistically significant. This enhanced activity of CBDA/CBGA-enriched fractions may be attributed to structural features of acidic cannabinoids, particularly the presence of a carboxyl group, which can enable additional hydrogen bonding and electrostatic interactions with enzyme active or peripheral sites, potentially increasing binding affinity compared to neutral cannabinoids. Collectively, these observations point to a possible synergistic or additive contribution of acidic cannabinoids in targeting AD-related enzymes. This finding challenges the traditional focus on decarboxylated cannabinoids and supports the therapeutic value of acid forms.

The different inhibitory profiles observed for AChE and BChE may be explained by structural differences between the two cholinesterases. Although both enzymes hydrolyze choline esters, BChE has a larger and more flexible active-site gorge, whereas AChE has a narrower and more restricted binding pocket. Therefore, cannabis-derived molecules, especially bulky or acidic cannabinoids such as CBDA and CBGA, may interact differently with each enzyme, leading to distinct inhibition profiles (Fang et al. 2011; Vitale et al. 2025; Puopolo et al. 2022; Mooko et al. 2022).

The detection of cannabinoid acids, including CBDA, CBGA, and THCA, in the active fractions provides a possible chemical context for the observed multi-target inhibitory profile. In our study, fractions containing acidic cannabinoids, particularly f5 and f6, showed strong inhibition of AChE and/or BACE1, while f5 also showed strong BChE inhibition. These findings are consistent with the study by Vitale et al. (Vitale et al. 2025) which reported that CBDA and CBGA can act as multi-target ligands against AChE, BChE, and BACE1 and showed beneficial effects in an Aβ-related model. Therefore, the findings of Vitale et al. support the biological relevance of cannabinoid acids and provide a rationale for further investigating CBDA/CBGA-containing fractions in the context of AD-related targets. However, because purified cannabinoids were not tested in the present study, the contribution of individual compounds remains to be confirmed.

**Fig. 10:**
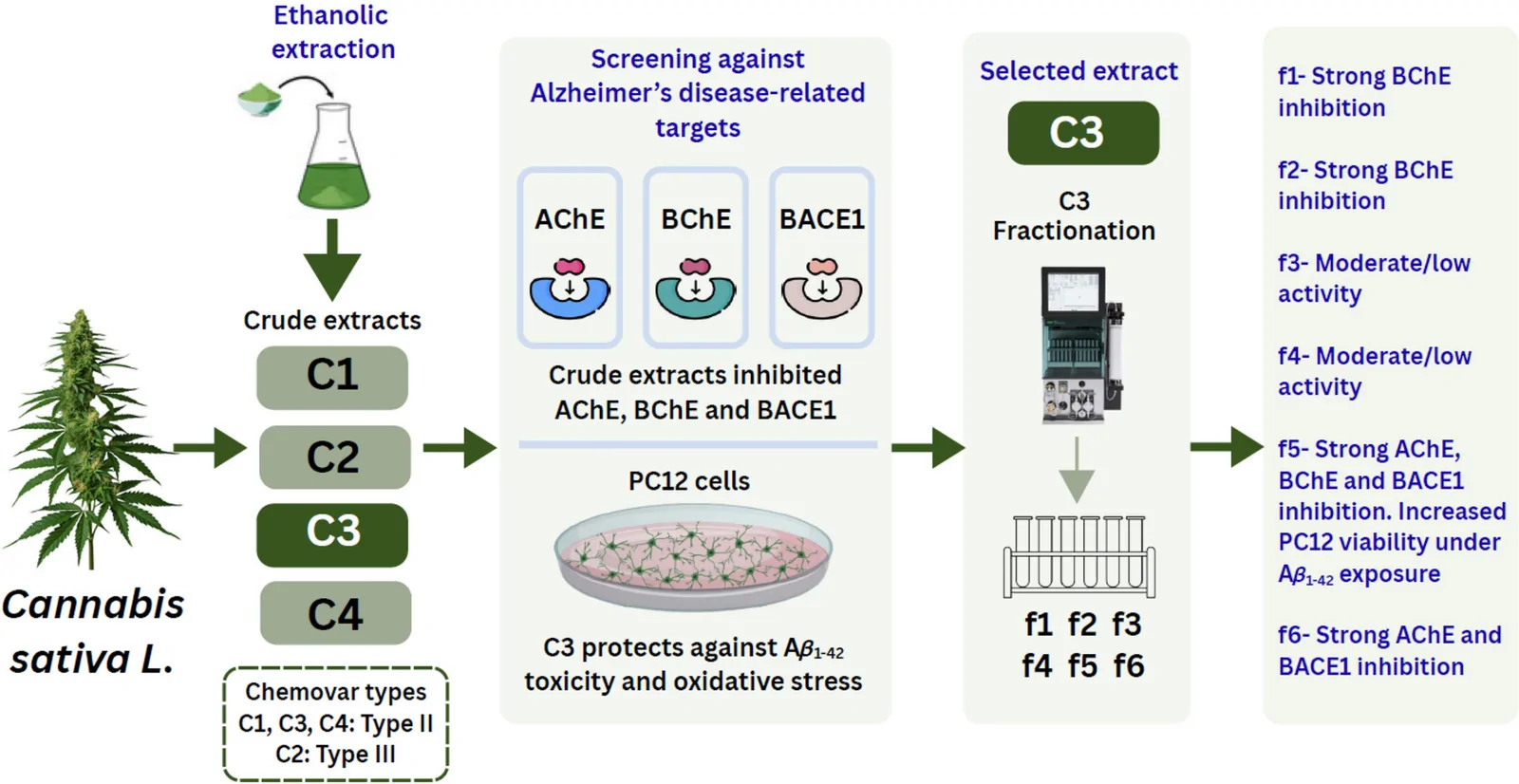
Graphical summary of the study workflow and main findings. Different chemovars of Cannabis sativa L. inflorescences were extracted with ethanol, and four crude extracts (C1–C4) were screened for activity against AD-related targets. Extract C3 showed broad in vitro activity, including inhibition of AChE, BChE, and BACE1, and cell-protective effects in PC12 cells under Aβ₁–₄₂ and oxidative stress conditions. C3 was further fractionated into six fractions, with f1 and f2 showing strong BChE inhibition, f5 showing strong AChE, BChE, and BACE1 inhibition with increased PC12 viability under Aβ₁–₄₂ exposure, and f6 showing strong AChE and BACE1 inhibition

As shown in Fig. 8A, fractions f5 and f6 exhibited significantly stronger inhibition of BACE1 compared to the crude extract C3 at the same concentration (25 μg/mL), achieving approximately 50% reduction in enzyme activity. This enhanced effect suggests that specific phytocannabinoids enriched in these fractions, particularly CBDA and CBGA, may play a central role in targeting BACE1. Furthermore, Fig. 8B demonstrates a clear dose-dependent pattern for fractions f5 and f6, where increasing concentrations corresponded with progressively higher levels of inhibition. Together, these findings highlight that the fractionation process not only preserved but amplified the inhibitory potential of C3 against BACE1, underscoring the importance of CBDA/CBGA-rich fractions as promising leads for further investigation in Alzheimer’s disease therapy (Fig. 10).

Taken together, the study highlights the promise of cannabis inflorescence extracts, especially from Type II and III chemovars and fractions, as promising in-vitro multi-target candidates for further investigation in AD related models. By combining cholinesterase inhibition, BACE1 inhibition, and neuroprotection against oxidative and amyloid stress, cannabis-derived compounds may offer a more comprehensive approach compared to current single-target drugs.

### Study limitations and future perspectives

Several limitations must be acknowledged: (i) the results are based on in vitro assays and require the incorporation of additional manipulations in cell lines as well as validation in animal models; (ii) the precise molecular mechanisms underlying the observed effects remain to be elucidated; (iii) the pharmacokinetics, blood–brain barrier permeability, and potential side effects of the active fractions are yet to be characterized; (iv) The presence of psychoactive cannabinoids, such as THC, and potential CYP450-mediated drug–drug interactions should be considered when evaluating future clinical translatability. Future studies should focus on evaluating the effects of C3 and its fractions in human iPSC-derived neuronal systems, including co-cultures with astrocytes or microglia. These studies should also investigate the underlying mechanisms by which the extracts and fractions protect neuronal cells against Aβ₁-₄₂-induced toxicity, including their effects on oxidative stress, mitochondrial function, and apoptotic pathways, and a correlation analysis and multivariate modeling between cannabinoid composition and biological activity, to identify the specific compounds or compound combinations responsible for the observed effects. Isolating and characterizing the most active phytocannabinoids, examining their synergistic interactions, and evaluating their efficacy in vivo. Given the complexity of AD pathology, cannabis-derived compounds may represent an emerging class of polypharmacological agents capable of addressing multiple disease pathways simultaneously.

## Supplementary Information

Supplementary Material 1.

## Data Availability

All data generated in this study are available from the corresponding author upon request.

## References

[CR1] Ahmad S, Ullah F, Ayaz M, Sadiq A, Imran M. Antioxidant and anticholinesterase investigations of Rumex hastatus D. Don: potential effectiveness in oxidative stress and neurological disorders. Biol Res. 2015;48(1):20. Available from: https://biolres.biomedcentral.com/articles/10.1186/s40659-015-0010-2. 10.1186/s40659-015-0010-2 PMC4381421 25857346

[CR2] Alzheimer’s Association. 2022 Alzheimer’s disease facts and figures. Alzheimer’s Dement. 2022;18(4):700–89. 10.1002/alz.12638 35289055

[CR3] Ayaz M, Junaid M, Ullah F, Sadiq A, Khan MA, Ahmad W, et al. Comparative chemical profiling, cholinesterase inhibitions and anti-radicals properties of essential oils from Polygonum hydropiper L: A Preliminary anti- Alzheimer’s study. Lipids Health Dis. 2015;14(1):141. Available from: http://www.lipidworld.com/content/14/1/141. 10.1186/s12944-015-0145-8 PMC4632677 26530857

[CR4] Bahji A, Breward N, Duff W, Absher N, Patten SB, Alcorn J, et al. Cannabinoids in the management of behavioral, psychological, and motor symptoms of neurocognitive disorders: a mixed studies systematic review. J Cannabis Res. 2022;4(1). Available from: 10.1186/s42238-022-00119-y. PMC8922797 35287749

[CR5] Balducci C, Forloni G. Novel targets in Alzheimer’s disease: A special focus on microglia. Pharmacol Res. 2018;130:402–13. Available from: https://linkinghub.elsevier.com/retrieve/pii/S1043661817313816. 10.1016/j.phrs.2018.01.017 29391235

[CR6] Cai H, Wang Y, McCarthy D, Wen H, Borchelt DR, Price DL, et al. BACE1 is the major β-secretase for generation of Aβ peptides by neurons. Nat Neurosci. 2001;4(3):233–4. Available from: https://www.nature.com/articles/nn0301_233. 10.1038/85064 11224536

[CR7] Cásedas G, Moliner C, Maggi F, Mazzara E, López V. Evaluation of two different Cannabis sativa L. extracts as antioxidant and neuroprotective agents. Front Pharmacol. 2022;13. Available from: https://www.frontiersin.org/articles/10.3389/fphar.2022.1009868/full. 10.3389/fphar.2022.1009868 PMC9513154 36176449

[CR8] Chaudhary A, Maurya PK, Yadav BS, Singh S, Mani A. Current Therapeutic Targets for Alzheimer’s Disease. J Biomed. 2018;3:74–84. Available from: http://www.jbiomed.com/v03p0074.htm.

[CR9] Cole SL, Vassar R. The Alzheimer’s disease Beta-secretase enzyme, BACE1. Mol Neurodegener. 2007;2(1):22. Available from: http://molecularneurodegeneration.biomedcentral.com/articles/10.1186/1750-1326-2-22. 10.1186/1750-1326-2-22 PMC2211305 18005427

[CR10] Doumar H, Mostafi H El, Elhessni A, Ebn Touhami M, Mesfioui A. Exploring the diversity of cannabis cannabinoid and non-cannabinoid compounds and their roles in Alzheimer’s disease: A review. IBRO Neurosci Reports. 2025;18:96–119. Available from: https://linkinghub.elsevier.com/retrieve/pii/S2667242124001192. 10.1016/j.ibneur.2024.12.011 PMC11763173 39866750

[CR11] Ellman GL, Courtney KDD, Andres V, Featherstone RM. A new and rapid colorimetric determination of acetylcholinesterase activity. Biochem Pharmacol. 1961;7(2):88–95. Available from: https://linkinghub.elsevier.com/retrieve/pii/0006295261901459. 10.1016/0006-2952(61)90145-9 13726518

[CR12] Fang L, Pan Y, Muzyka JL, Zhan CG. Active Site Gating and Substrate Specificity of Butyrylcholinesterase and Acetylcholinesterase: Insights from Molecular Dynamics Simulations. J Phys Chem B. 2011;115(27):8797–805. Available from: https://pubs.acs.org/doi/10.1021/jp112030p. 10.1021/jp112030p PMC3135420 21682268

[CR13] Faraji L, Nadri H, Moradi A, Bukhari SNA, Pakseresht B, Moghadam FH, et al. Aminoalkyl-substituted flavonoids: synthesis, cholinesterase inhibition, β-amyloid aggregation, and neuroprotective study. Med Chem Res. 2019;28(7):974–83. Available from: 10.1007/s00044-019-02350-4.

[CR14] Fleisher-Berkovich S, Sharon N, Ventura Y, Feinshtein V, Gorelick J, Bernstein N, et al. Selected cannabis cultivars modulate glial activation: in vitro and in vivo studies. J Cannabis Res. 2024;6(1):25. Available from: https://jcannabisresearch.biomedcentral.com/articles/10.1186/s42238-024-00232-0. 10.1186/s42238-024-00232-0 PMC11110427 38778343

[CR15] Flores-Sanchez IJ, Verpoorte R. Secondary metabolism in cannabis. Phytochem Rev. 2008;7(3):615–39. Available from: http://link.springer.com/10.1007/s11101-008-9094-4.

[CR16] Greene LA, Tischler AS. Establishment of a noradrenergic clonal line of rat adrenal pheochromocytoma cells which respond to nerve growth factor. Proc Natl Acad Sci. 1976;73(7):2424–8. Available from: https://pnas.org/doi/full/10.1073/pnas.73.7.2424. 10.1073/pnas.73.7.2424 PMC430592 1065897

[CR17] Greig NH, Utsuki T, Yu Q sheng, Zhu X, Holloway HW, Perry T, et al. A New Therapeutic Target in Alzheimer’s Disease Treatment: Attention to Butyrylcholinesterase. Curr Med Res Opin. 2001;17(3):159–65. Available from: http://www.tandfonline.com/doi/full/10.1185/0300799039117057. 10.1185/0300799039117057 11900310

[CR18] Jannat S, Balupuri A, Ali MY, Hong SS, Choi CW, Choi YH, et al. Inhibition of β-site amyloid precursor protein cleaving enzyme 1 and cholinesterases by pterosins via a specific structure−activity relationship with a strong BBB permeability. Exp Mol Med. 2019;51(2):1–18. Available from: https://www.nature.com/articles/s12276-019-0205-7. 10.1038/s12276-019-0205-7 PMC6372667 30755593

[CR19] Kostanda E, Khatib S. Biotic stress caused by Tetranychus urticae mites elevates the quantity of secondary metabolites, cannabinoids and terpenes, in Cannabis sativa L. Ind Crops Prod. 2022;176:114331. Available from: https://linkinghub.elsevier.com/retrieve/pii/S0926669021010967.

[CR20] Lewis M, Russo E, Smith K. Pharmacological Foundations of Cannabis Chemovars. Planta Med. 2018;84(04):225–33. Available from: http://www.thieme-connect.de. 10.1055/s-0043-122240 29161743

[CR21] Lima E, Medeiros J. Terpenes as potential anti-Alzheimer’s disease agents. Appl Sci (Basel). 2024. 10.3390/app14093898.

[CR22] Mahlberg PG, Kim ES. Accumulation of Cannabinoids in Glandular Trichomes of Cannabis (Cannabaceae). J Ind Hemp. 2004;9(1):15–36. Available from: http://www.tandfonline.com/doi/abs/10.1300/J237v09n01_04.

[CR23] Marsh DT, Shibuta M, Kato R, Smid SD. Medicinal cannabis extracts are neuroprotective against Aβ 1–42 ‐mediated toxicity *in vitro*. Basic Clin Pharmacol Toxicol. 2024. 10.1111/bcpt.14078. 39243211

[CR24] Mateos-Maces L, Chávez-Servia JL, Vera-Guzmán AM, Aquino-Bolaños EN, Alba-Jiménez JE, Villagómez-González BB. Edible Leafy Plants from Mexico as Sources of Antioxidant Compounds, and Their Nutritional, Nutraceutical and Antimicrobial Potential: A Review. Antioxidants. 2020;9(6):541. Available from: https://www.mdpi.com/2076-3921/9/6/541. 10.3390/antiox9060541 PMC7346153 32575671

[CR25] Mesulam MM, Guillozet A, Shaw P, Levey A, Duysen E., Lockridge O. Acetylcholinesterase knockouts establish central cholinergic pathways and can use butyrylcholinesterase to hydrolyze acetylcholine. Neuroscience. 2002;110(4):627–39. Available from: https://linkinghub.elsevier.com/retrieve/pii/S0306452201006133. 10.1016/s0306-4522(01)00613-3 11934471

[CR26] Mesulam M, Guillozet A, Shaw P, Quinn B. Widely Spread Butyrylcholinesterase Can Hydrolyze Acetylcholine in the Normal and Alzheimer Brain. Neurobiol Dis. 2002;9(1):88–93. Available from: https://linkinghub.elsevier.com/retrieve/pii/S0969996101904620. 10.1006/nbdi.2001.0462 11848688

[CR27] Mooko T, Bala A, Tripathy S, Kumar CS, Mahadevappa CP, Chaudhary SK, et al. Cannabis Sativa L. Flower and Bud Extracts Inhibited In vitro Cholinesterases and β-Secretase Enzymes Activities: Possible Mechanisms of Cannabis Use in Alzheimer Disease. Endocrine, Metab Immune Disord - Drug Targets. 2022;22(3):297–309. Available from: https://www.eurekaselect.com/191663/article. 10.2174/1871530321666210222124349 33618651

[CR28] Park SY, Kim HS, Cho EK, Kwon BY, Phark S, Hwang KW, et al. Curcumin protected PC12 cells against beta-amyloid-induced toxicity through the inhibition of oxidative damage and tau hyperphosphorylation. Food Chem Toxicol. 2008;46(8):2881–7. Available from: https://linkinghub.elsevier.com/retrieve/pii/S0278691508002779. 10.1016/j.fct.2008.05.030 18573304

[CR29] Peng Y, Jin H, Xue YH, Chen Q, Yao SY, Du MQ, et al. Current and future therapeutic strategies for Alzheimer’s disease: an overview of drug development bottlenecks. Front Aging Neurosci. 2023;15. 10.3389/fnagi.2023.1206572 PMC10438465 37600514

[CR30] Puopolo T, Liu C, Ma H, Seeram NP. Inhibitory Effects of Cannabinoids on Acetylcholinesterase and Butyrylcholinesterase Enzyme Activities. Med Cannabis Cannabinoids. 2022;5(1):85–94. Available from: https://karger.com/article/doi/10.1159/000524086. 10.1159/000524086 PMC9149358 35702400

[CR31] Raïch I, Lillo J, Rebassa JB, Griñán-Ferré C, Bellver-Sanchis A, Reyes-Resina I, et al. Cannabidiol as a multifaceted therapeutic agent: mitigating Alzheimer’s disease pathology and enhancing cognitive function. Alzheimers Res Ther. 2025;17(1):109. Available from: https://alzres.biomedcentral.com/articles/10.1186/s13195-025-01756-0. 10.1186/s13195-025-01756-0 PMC12090481 40394655

[CR32] Reis J, Cagide F, Valencia ME, Teixeira J, Bagetta D, Pérez C, et al. Multi-target-directed ligands for Alzheimer’s disease: Discovery of chromone-based monoamine oxidase/cholinesterase inhibitors. Eur J Med Chem. 2018;158:781–800. Available from: https://linkinghub.elsevier.com/retrieve/pii/S0223523418306214. 10.1016/j.ejmech.2018.07.056 30245401

[CR33] Savva K, Zachariou M, Kynigopoulos D, Fella E, Vitali MI, Kosofidou X, et al. Preliminary in vitro and in vivo insights of in silico candidate repurposed drugs for Alzheimer’s disease. Life (Basel). 2023;13(5):1–17. 10.3390/life13051095 PMC10221567 37240740

[CR34] Sharma K. Cholinesterase inhibitors as Alzheimer’s therapeutics (Review). Mol Med Rep. 2019. Available from: http://www.spandidos-publications.com/10.3892/mmr.2019.10374. 10.3892/mmr.2019.10374 PMC6625431 31257471

[CR35] Stine WB, Jungbauer L, Yu C, Ladu MJ. Preparing synthetic Aβ in different aggregation states. In: Methods in Molecular Biology. 2011. p. 13–32. Available from: http://link.springer.com/10.1007/978-1-60761-744-0_2. 10.1007/978-1-60761-744-0_2 PMC3752843 20967580

[CR36] Swenson K. Beyond the hype: a comprehensive exploration of CBD’s biological impacts and mechanisms of action. J Cannabis Res. 2025;7(1). Available from: 10.1186/s42238-025-00274-y. PMC12067965 40350443

[CR37] Tong Y, Bai L, Gong R, Chuan J, Duan X, Zhu Y. Shikonin Protects PC12 Cells Against β-amyloid Peptide-Induced Cell Injury Through Antioxidant and Antiapoptotic Activities. Sci Rep. 2018;8(1):26. Available from: https://www.nature.com/articles/s41598-017-18058-7. 10.1038/s41598-017-18058-7 PMC5758797 29311595

[CR38] Tumiatti V, Minarini A, Bolognesi ML, Milelli A, Rosini M, Melchiorre C. Tacrine Derivatives and Alzheimers Disease. Curr Med Chem. 2010;17(17):1825–38. Available from: http://www.eurekaselect.com/openurl/content.php?genre=article&issn=0929-8673&volume=17&issue=17&spage=1825. 10.2174/092986710791111206 20345341

[CR39] Vassar R. BACE1 inhibitor drugs in clinical trials for Alzheimer’s disease. Alzheimers Res Ther. 2014;6(9):89. Available from: https://alzres.biomedcentral.com/articles/10.1186/s13195-014-0089-7. 10.1186/s13195-014-0089-7 PMC4304279 25621019

[CR40] Vitale RM, Morace AM, D’Errico A, Ricciardi F, Fusco A, Boccella S, et al. Identification of Cannabidiolic and Cannabigerolic Acids as MTDL AChE, BuChE, and BACE-1 Inhibitors Against Alzheimer’s Disease by In Silico, In Vitro, and In Vivo Studies. Phyther Res. 2025;39(1):233–45. Available from: https://onlinelibrary.wiley.com/doi/10.1002/ptr.8369. 10.1002/ptr.8369 PMC11745148 39510547

[CR41] Wang L, Cao J, Shi Z, Fan W, Liu H, Deng J. Experimental study on the neurotoxic effect of β-amyloid on the cytoskeleton of PC12 cells. Int J Mol Med. 2018. 10.3892/ijmm.2018.3467. PMC5846656 29436599

[CR42] Wiatrak B, Kubis-Kubiak A, Piwowar A, Barg E. PC12 cell line: cell types, coating of culture vessels, differentiation and other culture conditions. Cells. 2020. 10.3390/cells9040958. PMC7227003 32295099

[CR43] Xie D, Deng T, Zhai Z, Sun T, Xu Y. The cellular model for Alzheimer’s disease research: PC12 cells. Front Mol Neurosci. 2023;15. Available from: https://www.frontiersin.org/articles/10.3389/fnmol.2022.1016559/full. 10.3389/fnmol.2022.1016559 PMC9846650 36683856

[CR44] Yao Q, Jiang K, Lin F, Zhu T, Khan NH, Jiang E. Pathophysiological association of Alzheimer’s disease and hypertension: a clinical concern for elderly population. Clin Interv Aging. 2023;18(May):713–28. 10.2147/CIA.S400527 PMC10167960 37181536

[CR45] Zhang D, Lv JT, Zhang B, Sa RN, Ma BB, Zhang XM, et al. Molecular insight into the therapeutic promise of xuebijing injection against coronavirus disease 2019. World J Tradit Chinese Med. 2020;6(2):203. Available from: https://journals.lww.com/10.4103/wjtcm.wjtcm_22_20.

