# Supplementary material for: Cannabis flower extracts as a multi-target agent in Alzheimer’s disease: Inhibition of AChE, BChE, and BACE1 with cell-protective effects in a PC12 cell model
Source: J Cannabis Res. 2026 Jul 31;8:113. doi: 10.1186/s42238-026-00479-9 (PMC13711765; doi:10.1186/s42238-026-00479-9)
Supplement: Supplementary file 1 [file 42238_2026_479_MOESM1_ESM.docx]

**Figure S1.** LC-MS/MS Chromatogram of cannabinoids analytical standards. (14 analytes, 1.25 ppm)

**Table S1.** Cannabinoid standards: determination of molar mass, chemical formula, retention time (RT) and R² in LC-MS/MS Analysis.

CBDA

**A**

CBCA

THCA

**B**

CBD

CBDA

THC

**Figure S2. Representative total ion chromatograms (TICs) of extract C1 acquired by LC–MS.** Chromatograms were recorded in negative ionization mode (A) and positive ionization mode (B). The x-axis represents retention time (min), and the y-axis represents base peak intensity. Major detected ions are labeled by their corresponding *m/z* values.

CBCA

CBDA

**B**

**A**

CBD

CBDA

**Figure S3. Representative total ion chromatograms (TICs) of extract C2 acquired by LC–MS.** Chromatograms were recorded in negative ionization mode (A) and positive ionization mode (B). The x-axis represents retention time (min), and the y-axis represents base peak intensity. Major detected ions are labeled by their corresponding *m/z* values.

CBGA

CBNA

CBCA

THCA

CBDA

**A**

CBCA

THCA

THC

CBD

CBDA

**B**

**Figure S4. Representative total ion chromatograms (TICs) of extract C3 acquired by LC–MS.** Chromatograms were recorded in negative ionization mode (A) and positive ionization mode (B). The x-axis represents retention time (min), and the y-axis represents base peak intensity. Major detected ions are labeled by their corresponding *m/z* values.

CBCA

THCA

CBNA

CBGA

**A**

CBDA

CBCA

THCA

THC

**B**

CBDA

CBD

**Figure S5. Representative total ion chromatograms (TICs) of extract C4 acquired by LC–MS.** Chromatograms were recorded in negative ionization mode (A) and positive ionization mode (B). The x-axis represents retention time (min), and the y-axis represents base peak intensity. Major detected ions are labeled by their corresponding *m/z* values.

CBDA

CBGA

CBGA

CBDA

**A**

CBDA

CBGA

**B**

**Figure S6. Representative total ion chromatograms (TICs) of extract F5 acquired by LC–MS.** Chromatograms were recorded in negative ionization mode (A) and positive ionization mode (B). The x-axis represents retention time (min), and the y-axis represents base peak intensity. Major detected ions are labeled by their corresponding *m/z* values.
